# Gene mutations responsible for primary immunodeficiency disorders: A report from the first primary immunodeficiency biobank in Iran

**DOI:** 10.1186/s13223-016-0166-5

**Published:** 2016-12-02

**Authors:** Saba Sheikhbahaei, Roya Sherkat, Dirk Roos, Majid Yaran, Somayeh Najafi, Alireza Emami

**Affiliations:** 1 Acquired Immunodeficiency Research Center, Isfahan University of Medical Science, Khoram St, Isfahan, Iran; 2 Sanquin Blood Supply Organization, and Landsteiner Laboratory, Academic Medical Centre, University of Amsterdam, Amsterdam, The Netherlands

## Abstract

**Background:**

Primary immunodeficiency (PID) is a heterogeneous group of inheritable genetic disorders with increased susceptibility to infections, autoimmunity, uncontrolled inflammation and malignancy. Timely precise diagnosis of these patients is very essential since they may not be able to live with their congenital immunity defects; otherwise, they could survive with appropriate treatment. DNA biobanks of such patients could be used for molecular and genetic testing, facilitating the detection of underlying mutations in known genes as well as the discovery of novel genes and pathways.

**Methods:**

According to the last update of the International Union of Immunological Societies (IUIS) classification, patients are registered in our biobank during a period of 15 years. All patients’ data were collected via questionnaire and their blood samples were taken in order to extract and protect their DNA content.

**Results:**

Our study comprised 197 patients diagnosed with PID. Antibody deficiency in 50 patients (25.4%), phagocytic defect in 47 patients (23.8%) and combined immunodeficiency with associated/syndromic feature in 19 patients (9.6%) were the most common PID diagnoses, respectively. The most common variant of PID in our study is common variable immunodeficiency, which accounted for 20 cases (10.1%), followed by chronic mucocutaneous candidiasis in 15 patients (7.9%) and congenital neutropenia in 13 patients (7%). Mean age at onset of disease was 4 years and mean age of diagnosis was 9.6 years. The average diagnostic delay was 5.5 years, with a range of 6 months to 46 years. Parental consanguinity and history of PID in family were observed in 70.2 and 48.9% of the patients, respectively. The majority of PID patients (93.3%) were from families with low socioeconomic status.

**Conclusion:**

This prospective study was designed to establish a PID Biobank in order to have a high quality DNA reservoir of these patients, shareable for international diagnostic and therapeutic collaborations. This article emphasizes the need to raise the awareness of society and general practitioners to achieve timely diagnosis of these patients and prevent current mismanagements.

## Background

Primary immunodeficiency (PID) refers to a complex genetic group of disorders characterized by defects in the immune system, resulting in high susceptibility to various infections [1]. Publications on PID patients have improved our knowledge that at present about 250 genes are involved in distinct immunodeficiency disorders [2]. A report from the Iranian Primary Immunodeficiency Registry (IPIDR) established the incidence of PID at 13 per 1,000,000 population, and a mortality rate of 18.7%, approximately similar to the global mortality rate. Although significant advances in the identification of PIDs have been made, its prevalence is underestimated owing to lack of awareness of the public and general practitioners [3, 4].

Since most PIDs are inherited in an autosomal recessive pattern, consanguineous marriage leads to a higher rate of their prevalence [5, 6]. Frequency of inter-family marriage in Muslim societies such as Iran is higher than in non-Muslim societies [7–9]. Data from the IPIDR revealed that 63% of the PID patients have consanguineous parents [4].

Biobanks are designed to store the samples and data from patients willing to participate in biomedical research. It also provides accessibility to patients’ samples for long-term evaluations, appropriate diagnosis and treatment [10]. It enables setting up links between medical centers around the world for research and therapeutic purposes, which would be particularly helpful for rare diseases [11]. In this study, we introduce a Biobank for PID patients (PIDB) with the aim of collecting and preserving sensitive data and biological samples. PIDB permits the assessment of the proportion of the affected individuals by genome sequencing and determining undiagnosed types of PID.

## Necessity of creating a biobank

Inadequate sample availability and poor biospecimen quality are the limitations of the case–control and cohort studies, particularly in the field of genetic disorders [12, 13]. Without a data and specimen bank, lots of time and material are wasted in each cross-sectional study. These limitations could be overcome by establishing a comprehensive biobank and database for affected patients. Storage of blood samples accelerates the process of laboratory investigations and offers the opportunity of studying several specimens simultaneously. Human DNA, a stable molecule containing genetic information, is extensively used for research purposes. The benefit of setting up a DNA bank is to overcome diagnostic limitations in our country by expansion of international collaborations. The collected samples could be distributed across borders for basic and clinical research projects resulting indefinite diagnosis [14].

## Methods

### PIDB management and funding

Our PID biobank is constructed under the support of our Immuno-Deficiency Research Center (IDRC) and managed by the head of this research center. Isfahan Immunodeficiency Association is a private charity association, which financially supports this project. It will be explained further that samples are primarily prepared in our center and are sent to the partner centers across the world. The Academic research collaboration agreements have been designed to carry out the genetic studies without charge.

### PIDB database

This retrospective study comprised patients with the diagnosis of PID who are referred to the clinical immunology clinics in Isfahan or are hospitalized in Alzahra hospital for receiving IVIg and other parental therapies. We used the criteria of European Society for Immunodeficiencies (ESID) and the International Union of Immunological Societies (IUIS-2014) for diagnosis of PID, from 2000 to 2015 [15, 16]. The family members of the patients who are suspected of any kind of PID or even presenting atypical manifestations are included in our survey. In particular, the individuals born to consanguineous parents took priority in being investigated. All individuals were provided with an information sheet in which the purposes of creating the PIDB and possible further research were written. Each patient who agreed to participate in the project was given a unique code, which served as the label for the specimen tube and the data sheet. Data were collected through questionnaires including detailed demographic information, socioeconomic status, parental consanguinity, family history of PID, number of deaths due to PID, first clinical manifestations of disease, history of having recurrent infections, history of autoimmune disease, history of atopy, laboratory results, treatments, information from stored medical documents and interviews with patients. Data were collected in Excel database and converted for analysis by the SPSS statistical software package version 16. The average maximum and minimum values were used for quantitative variables. ANOVA was utilized to compare quantitative variables for more than 2 groups. Pearson’s chi^2^ test was used to compare nominal/ordinal variables among groups. A p value lower than 0.05 was considered statistically significant.

### PIDB sampling

After the inform consent had been signed, a 10-ml blood sample of each patient was taken in a tube with anticoagulant for the extraction of DNA and RNA. The process of extraction was carried out with calibrated instruments according to standard protocols. It has been shown that adding citrate as anticoagulant yields higher quality RNA and DNA; however, Ethylene diamine tetra-acetic acid (EDTA)-coated collection tubes are also suitable for extraction of DNA and protein but may show some unwanted side effects [17]. Peripheral blood mononuclear cells (PBMC) were isolated from the buffy coat by Ficoll-Hypaque density gradient. All specimens were processed and archived immediately after sample collection to avoid potential degradation, because a time difference between sampling and cryopreservation of the biomolecules of more than 24 h could affect the quality of samples. Freshly obtained PBMC were processed for DNA and RNA extraction with the High Pure PCR template preparation Kit, and the cDNA that was synthesized with reverse transcriptase enzyme, were all kept at −70 °C. It has been established that DNA is stable at 4 °C for weeks, at −20 °C for months and at −70 °C for years, so −70 °C is a suitable temperature for long-term stable storage of DNA. However, there is some evidences that RNA may be damaged over 5 years of maintenance at this temperature. All steps of sample preparation were performed by a trained team of nurses and technicians. The quality of samples in biobanks should be guaranteed, thus the used protocol provided rigorous quality assurance and control. We used spectrophotometry for rapid evaluation of the yield and purity of the extracted DNA and RNA. An OD 260/280 ratio higher than 1.8 was considered an indicator of acceptably pure DNA/RNA, relatively free of protein. Since spectrophotometry does not reflect the integrity of the genomic content, agarose gel electrophoresis was applied as well. Concentration and yield was determined by comparing the sample DNA intensity to that of a DNA quantitation standard. To ensure that anonymity was preserved, specimens including DNA, RNA and cDNA were labeled with their identification codes.

### PIDB role in medical research

Creation of a PIDB not only provides an opportunity to secure genetic information for further molecular studies but also describes epidemiological data on different types of PID [18]. This biorepository plays a significant role in the recognition of known gene mutations or new gene mutation discoveries by proper storing and processing of the collected samples. A PIDB is a resource of large amounts of DNA, which is especially beneficial in genetic analysis of families with complex pedigrees resulting from consanguineous marriages.

## PIDB ethical issues

### Informed consent

Informed consent is one of the major principles of ethics that should be considered in DNA biobank studies as well as other research surveys in which specimens are obtained through intervention [19]. Consent allows patients to decide whether they are interested in participating in a study with a given sample of their body. All individuals have to be well informed about the purpose of the study and the associated risks and benefits, and then a voluntary consent with preserved rights of patients will be obtained. All participants are allowed to withdraw the consent at any time in the study. Since biobank samples are not prepared just for one study, consent from the donors has to be obtained for all further research except if the patients have agreed to continue with the usage of their samples under a broad consent form initially. We preferred to seek informed consent for one study; however, this requires re-contact of patients for any new purpose that has not been declared in the primary form. Frequent contact with the donor assures patients that the process of diagnosing their disease is continuing and has not been stopped because there has not been any noticeable achievement. Since a dead participant cannot be re-contacted, we agreed with the authors that if obtaining re-consent is impossible, it would be acceptable to re-use the samples without consent [20–22].

Most biobank studies do not contain samples of children, because they do not always understand the purpose of research studies, they are not easily accessible, obtaining samples from children requires more skills and also because they suffer relatively more than adults. Some authors believe that sharing data on children should not be allowed ethically until they reach adulthood and have the right to decide whether they want to be involved in the investigation [23]. However, other authors argue that the data will expire and the individual himself/herself cannot take the advantage [24]. In our study, which mainly comprised children, parents are the only eligible ones who could sign the informed consent on their behalf. This investigation was approved by the Medical Ethics Committee of the Isfahan University of Medical Sciences under approval no. 290130.

### Participants’ privacy

One of the participants’ concerns in blood donation is confidentiality about their personal information [25]. Biobanks contain genetic information about each individual with a specific phenotype. By using anonymous samples, the link between the lab and personal data is broken and just longitudinal epidemiologic results can be achieved. Best solution is coding data, which guarantees the protection and is acceptable in standard research experiments [26–28].

We established clear policies to secure patients’ privacy, such as identifying different levels of access to the data by the employees of PIDB and encoding of biospecimens and data [29].

There are some issues on the exchange of data and sharing of databases in international collaborations. In this situation, the risk of confidentiality breach increases, and this is the reason why most researchers are reluctant to have the data sets shared [30]. Our international collaboration is extremely encouraged, even by the patients, as it is not only reaching research goals but also gives aid in diagnosis of their disease. Hence, we took measures to maximize the patients’ privacy by transmitting coded data.

In this type of research, in which genetic biobank of a population of rare diseases is targeted, patients have to be notified on the concrete genetic findings relevant to their medical therapies once the final results have been prepared. The duty of informing patients equally and immediately after their definite diagnosis is an obligatory principle.

## Partnership

Identification of the genetic basis of a primary immunodeficiency disease requires sufficient number of cases and availability of high technologies to discover the molecular origin of genetic disorders. Following the establishment of a PID biobank, a memorandum of understanding was signed between the Isfahan University of Medical Sciences and the Hanover Medical School and Ludwigs Maximillians University of Munich, Germany. Academic partnerships with France and Sweden for research and clinical collaborations were developed afterwards. As stated in the method section, DNA, RNA and cDNA samples from patients were prepared in accordance to the type of information to be obtained and the level of sensitivity necessary. One of the techniques for gene expression profiles—RNA sequencing, exome sequencing and next generation sequencing—were applied. RNA transcriptome sequencing mostly focused on gene expression profile and also detects alternative splicing events but apart from being costly and time consuming, its usage would be limited in genes with relatively low expression. In exome sequencing, because DNA is targeted, there is no difficulty in detecting low-expressed genes.

## Results

The biobank of PID consists of 197 samples, 121 male and 76 female, from 2000 to 2015. The classification of registered patient according to IUIS is presented in Table 1. Antibody deficiency and phagocytosis defects, including number and/or function, were the most common groups of PID disease, with 50 patients and 47 patients, respectively. Other subcategories of PID were as follows: combined immunodeficiency with associated/syndromic feature 19 cases, innate immunity disorders and auto inflammatory diseases each 13 cases, combined immunodeficiency 12 cases, immune dysregulation 7 cases and complement deficiency 3 cases. 34 patients are presented with different manifestations of primary immunodeficiency diseases but are not yet categorized in a specific group. CVID^1^ was the most common disorder, with 20 patients. CGD^2^ (n = 14), CMC^3^ (n = 13), and MSMD^4^ (n = 12) were also common PIDs defined in our registered patients.

**Table 1:** Distribution of PID cases registered in first primary immunodeficiency biobank in Iran according to IUIS classification, rate of consanguinity, family history of PID, socioeconomic status, age at onset and diagnosis and diagnosis delay

Disorders	Number	Sex		Consanguinity	PID in family	Socioeconomic status		Age at onset	Age at diagnosis	Diagnosis delay
		M	F			High	Low			
CID with syndromic/associated feature	19 (9.6%)	78.9%	21%	83.3% 16	23.1%	5.6%	94.4%	2.5 (0.1–9)	7.1 (0.5–21)	3.8 (0.5–16)
AT	8									
WAS	2									
HIES	7									
DiGeorge anomaly	1									
Comel–Netherton syndrome	1									
CID	12 (6%)	66.6%	33.3%	75%	83.3%	0%	100%	2 (0.3–0.5)	7.4 (1–15)	5.4 (0.6–12)
SCID	2			9						
ADA def	3									
MST1 def	3									
MHC II def	1									
CD40 def	2									
Ab deficiency	50 (25.4%)	76%	24%	64% 32	44%	4%	96%	5.3 (0.1–31)	11 (1–51)	5.6 (0.6–46)
CVID	20									
HIGM	2									
THI	1									
Bruton	10									
BTK deficiency	3									
CD-40 ligand def	1									
SIgAD	3									
NFKB2 def	1									
SADNI	12									
Congenital defect of Phagocytosis	47 (23.8%)	51%	48.9%	63.8% 30	38.3%	12.7%	87.2%	3 (0.1–30)	7.9 (1–31)	5 (0.5–23)
CGD	14									
MSMD	12									
Cyclic neutropenia	8									
Severe congenital neutropenia	7									
LAD	7									
Innate immunity defect CMC	13 (6.6%)	53.8%	46.2%	55.6% 7	80%	0%	100%	5.5 (0.3–20)	12.6 (2–26)	7.1 (1–19)
Autoinflammatory	13 (6.6%)	61.5%	38.5%	46.1%6	69.2%	15.4%	92.3%	5.5 (1–10)	11.7 (4–27)	6.2 (1–22)
FMF	8									
Disease of immune dysregulation	7 (3.5%)	42.9%	57.1%	50%	40%	0%	100%	6 (2–13)	11.1 (4–19)	5.1 (2–12)
IPEX	2									
APECED	2									
Chediack Higashi	3									
Complement deficiency C1 inhibitor deficiency	3 (1.5%)	33.3%	66.6%	33.3% 1	0%	0%	100%	5.5 (5–6)	8.5 (8–9)	3 (3–3)
Unknown	34 (17.2%)	55.9%	44.1%	78.1% 25	62.1%	6.5%	93.5%	3.7 (0.1–12)	–	–
Total/average	197 (100%)	121 (61.4%)	76 (38.6%)	71% 122	48.9%	6.7%	93.6%	4.0 (0.1–31)	9.6 (0.5–51)	5.5 (0.5–46)

Our survey resulted in a molecular genetic diagnosis for 33 out of 197 patients. Another 160 patients who were suspected of having PIDs according to clinical symptoms did not yet receive a genetic diagnosis. No mutation was detected in 4 cases by using targeted gene panel—which focuses on a set of relevant candidate genes with known diagnostic yield. Thus, the samples are under further investigations by applying whole exome sequencing (WES). Detailed genetic diagnosis of patients, including the affected gene, the mutation, the PID subclass, the molecular genetic test by which the diagnosis was carried out and confirmation by Sanger sequencing are listed in Table 2. Most of the patients presented typical clinical manifestations of known PIDs, so that they were likely to be diagnosed based on a classical approach. Identifying gene mutations in the Iranian PID population resulted in 2 novel mutation discoveries, JAGN1^5^ and STK4^6^ deficiencies, which are the causes of a type of CVID and CID, respectively [31, 32].

**Table 2:** Detailed genetic analysis of some PID patients from PIDB in Iran

ID	Validated by Sanger	Method	Disease	Gene affected	Mutation	Comment
P1	No	NGS	Not known	CDX1 (exone 3)	G > A, p. E209D	Heterozygous gene mutation in CDX1 (exone 3)
P2	Yes	NGS	Ab def	NFKB2	Not reported yet	Frame shift mutation in NFKB2
P3	No	CVID panel, WES	UI*	–	–	No deleterious mutation diagnosed in the CVID chip
P4	No	Hyper IgE panel, WES	UI*	–	–	No promising data have been detected
P5	Yes	NGS,	Bruton	BTK	c.1783T > C, p.Y551H	Missense mutation in BTK
P6	No	CVID panel, WES	UI*	–	–	No deleterious mutation diagnosed in the CVID chip
P7	Yes	NGS,	Netherton syndrome	SPINK5	c.A/−, p.N755Mfs*27	Homozygous nonsense mutation in SPINK5; (Netherton)
P8(A)	Yes	Primary Ab def panel	Bruton	BTK	c.115T > C, p.Y39H	
P9(A)	Yes	Primary Ab def panel	Bruton	BTK	c.115T > C, p.Y39H	
P10	Yes	NGS	CID	PIK3CD	c.3061G > A,p.E1021 K	Heterozygous mutation in the PIK3CD gene
P11	Yes	NGS	CID (MHC II def)	RFXANK	c.438 + 5G > A	RFXANK/BLS (Bare Lymphocyte Syndrome)
P12	Yes	WES	Phagocytic defect	STAT 1	c.1945C > T, p.R649C	Heterozygous gene mutation in STAT 1
P13	No	MSMD panel, WES	UI*	–	–	No mutation in MSMD genes
P14	No	WES	AT	ATM gene (exon 39)	c.5623C > T,p.Arg1875	Rare homozygous variants here; mutation in exon 39 of ATM gene in homozygous form ATM gene
P15	No	NGS	SCID	PTPRC(CD45RO)	c.2539A > G	CD45RO deficiency
P16 (B)	Yes	WES	CGD	NCF1	Completely absent	Complete lack of the NCF1 gene that encodes p47-phox, a component of NADPH oxidase enzyme. Only NCF1 pseudogenes were found
P17 (B)	Yes	WES	CGD	NCF1	Completely absent	Complete lack of the NCF1 gene that encodes p47-phox, a component of NADPH oxidase enzyme. Only NCF1 pseudogenes were found
P18	Yes	NGS	WAS	WAS	non-reported	Non-reported mutation in exon 8 was found as hemizygous (WAS)
P19	YES	WES	CID (CD40 def)	TNFRSF13B	IVS3 + 25 A > C	Heterozygote mutation in TNFRSF13B)
P20 (C)	Yes	WES	LAD	ITGB2	1670 G > C	Mutation in ITGB2 gene (LAD I)
P21 (C)	Yes	WES	LAD	ITGB2	1670 G > C	Mutation in ITGB2 gene (LAD I)
P22	Yes	WES	CGD (X-linked)	CYBB (exon 3)	c.252 + 5 G > C	Mutation in exon 3 of CYBB gene on Ch-X
P23 (D)	Yes	WES	FMF	MEFV	c.2282G > A:p.R761H/c.2040G < C:p.M680H	Homozygous mutation in MEFV gene
P24 (D)	Yes	WES	FMF	MEFV	c.2040G < C:p.M680H	Heterozygous mutation in MEFV gene
P25 (D)	Yes	WES	FMF	MEFV	c.2282G > A:p.R761H	Heterozygous mutation in MEFV gene
P26 (D)	Yes	WES	FMF	MEFV	c.2282G > A:p.R761H	Heterozygous mutation in MEFV gene
P27 (D)	Yes	WES	FMF	MEFV	c.2040G < C:p.M680H	Heterozygous mutation in MEFV gene
P28	Yes	WES	SCN	JAGN1	c.59G > A,p.Arg20Glu	Homozygous mutation in JAGN1 gene
P29	Yes	WES	SCN	JAGN1	c.40G > A, p.Gly14Ser	Homozygous mutation in JAGN1 gene
P30 (E)	Yes	SNP mapping array	MST1 def	STK4	c.G750A, p.W250X	Homozygous stop codon mutation in exon 7 of the gene *STK4*
P31 (E)	Yes	SNP mapping array	MST1 def	STK4	c.G750A, p.W250X	Homozygous stop codon mutation in exon 7 of the gene
P32 (E)	Yes	SNP mapping array	MST1 def	STK4	c.G750A, p.W250X	Homozygous stop codon mutation in exon 7 of the gene STK4
P33	No	CVID panel	Ab def (CD40L def)	TNFSF5	c.521A > G	Homozygous mutation in TNFSF5 gene
P34 (F)	Yes	NGS	CGD	CYBA	c.388C > T; p.G130X	Homozygous non-sense mutation in CYBA-encoding p22-phox:CAG codon change for Gln-130 → TAG stop codon
P 35 (F)	Yes	NGS	CGD	CYBA	c.388C > T; p.G130X	Homozygous non-sense mutation in CYBA-encoding p22-phox:CAG codon change for Gln-130 → TAG stop codon
P 36 (H)	Yes	WES	CGD	CYBB	C730delT; p.Cys244ValfsX10	One base-pair deletion in CYBB, the X-ch linked gene encoding gp91-phox
P 37 (H)	Yes	WES	CGD	CYBB	C730delT; p.Cys244ValfsX10	One base-pair deletion in CYBB, the X-ch linked gene encoding gp91-phox

* UI Under Investigation

Patients with the same letter in parentheses after their IDs are from one family

## Data analysis

Our data analysis revealed that the median age of the registered patient is 15 years and 10 months, with a wide range from 2 to 58 years. Median age at the onset of disease in different types of PID is 2.5 years, ranging from infancy to 58 years. We observed first signs and symptoms of PID in 34.8% of patients under 1 year and 50% under 2.5 years. Only 12 cases showed first clinical manifestations of PID after the age of 10 years. It is shown that median age of onset among patients with different types of PID has a minimum of 1 and a maximum of 5.5 years, which is attributed to phagocytic defect and complement deficiency, respectively.

The median age of diagnosis was 7 years, with a range of 7 months to 51 years. Phagocytic defects in number/function accounts for the lowest mean age of diagnosis, which is 4 years, while innate immunity defect is diagnosed at older age, with a median age of 14 years and 7 months. Diagnostic delay varied from 6 months to 46 years, with a median of 3 years. The median period of 4.5 years of diagnosis delay was the highest in patients with auto inflammatory disease, and 2 years was the lowest diagnostic delay seen in antibody deficiency patients. 20.3% of patients (cases) were diagnosed a year after the onset of disease and diagnosis was made later than 4 years after onset in about 38% of patients.

Ethnicity and religion were assessed in all patients. Almost all patients were Muslims, and 97% of patients were Persian. Two patients were Arab, 1 Turkish and 1 Kurdish.

CID^7^, combined immunodeficiency with associated/syndromic feature and antibody deficiency was observed threefold more frequently in males (M: F; 62:19). However, other PIDs affected males and females equally.

Among the different types of PID, parental consanguinity did not differ statistically. 100 and 22 patients had consanguineous parents, and 63.9% were children of first-cousin marriages. Highest rate of consanguinity was seen in combined immunodeficiency with associated/syndromic feature and CID^7^. History of PID in the family occurred in 48.9% of patients and was significantly higher in patients with CID^7^and innate immunity defects. Forty-three patients belonged to 16 kind reds, and gene mutations responsible for the PIDs were determined in 21 individuals from 8 families. 100 and 75 patients were from families with low socioeconomic status, and no significant difference was seen in socioeconomic status between different groups of PID (Table 1).

## Discussion

In the last decade, much effort has been devoted to establish biobanks for different research purposes. It has been a long-standing need to create a biobank for PID patients in Iran. Construction of such a biobank as a resource center of data and genome contents facilitates identification of genetic defects underlying different types of PID, in spite of the generation of descriptive statistics. We created a PIDB in order to stop the ad-hoc research studies, which may not have standard quality of sampling, processing and storage.

In our population, 30 different PIDs were diagnosed and categorized into 8 main groups. This is a preliminary report of our PID biobank and database. Our study showed that antibody deficiency is the most common group of PIDs in Iran, which is consistent with other studies [29, 33–37]. The proportion of patients with antibody deficiency in total is almost similar to previous reports from Iran but lower than the last report from ESID (26 vs. 57%) [4, 37]. Congenital defect of phagocyte number and/or function is the second most predominant PID in our study, similar to studies from France, Malaysia, Korea, USIDNET^8^, Iran and Iceland [4, 36–39], and is in contrast with studies from other registries that reported combined immunodeficiency with associated/syndromic feature as the second common one [33, 35, 39, 40]. Phagocyte defect involved 23.9% of our patients and 42% of PIDs in Oman [41]. However, much lower frequency has been observed in ESID, UK, Turkey and Spain [35, 39, 42]. Combined immunodeficiency with associated/syndromic feature was the third prevalent PID, which accounts for 10% of patients in accordance with studies from France, Malaysia and UK. This group of PID was formerly known as well-defined syndromes, but it has been changed into combined immunodeficiency with associated/syndromic feature in the updated IUIS. Innate immunity defects and auto inflammatory diseases are followed by combined immunodeficiency and disease of immune dysregulation. Complement deficiency was the least prevalent subcategory in our study and also in the other surveys [39, 43]. CVID^1^ was the most frequent disorder in most studies [34, 39], which is in contrast with the last update of IPIDR that indicated SCID^9^ as the most common disorder in Iran [4].

It was not an unexpected finding that PID in males is more frequent than females (1.7:1), but we found a significant difference in sex distribution in CID^7^, combined immunodeficiency with associated/syndromic feature, antibody deficiency and auto inflammatory disorders compared to other PID groups. The fact that males are more affected by PID is partially related to known X-linked disorders. Our registry included only 10 BTK^10^ deficiencies, 1 CD-40 ligand deficiency, 2 WAS^11^ and 1 IPEX^12^ patients. An important finding is that, besides these known X-linked diseases, there is still a significant difference in gender distribution in the mentioned groups. This finding may suggest the presence of undiscovered X-linked patterns of inheritance in PID groups among our population. Few studies evaluated consanguinity in PID families. Our study is the first study to analyze consanguinity among different groups of PIDs, but no significant difference was observed. Recent studies and other registries from Islamic countries and also from Germany showed a higher rate of consanguineous marriage in parents of PID patients compared to the rate reported from the UK and Turkey [35, 42]. In our PID population, consanguinity was mostly observed in patients with CID^5^ and CID with associated/syndromic feature. Our study evaluated the history of any known/suspicious case of PID in family members of the patients, which was more frequent than in another report from Iran (48.9 vs. 28.9%) [4]. It is probably due to the consideration of positive family history of PID in relatives of patients who did not yet have a definite genetic diagnosis.

Diagnostic delay in our study (5 years and 6 months) was much higher than that of other studies. This may be due to ignorance of symptoms and mismanagements of patients by inept physicians. The clinical evidence for this fact is that 22 patients suffer from bronchiectasis (11.7%). The delay from onset of symptoms to diagnosis was shorter in CID^7^ with syndromic/associated feature, because these patients have significant presentations, distinctive features in childhood or intrauterine defects including coarse faces, microcephaly, mental retardation, dwarfism, and IUGR, among others which cannot be neglected. Our registry also reports 7 deaths due to primary immunodeficiency disease that can be partly related to delayed or undiagnosed type of disease. As patients with PID are struggling with various recurrent infections, lifelong, and no definite treatment is known except bone marrow transplantation, in some cases, they just receive therapies in order to control signs and symptoms. Consequently, these patients visit their clinical immunologists for follow up and renew their drugs frequently. Our last update revealed that 61 patients were not followed in the previous year, and we are unaware of their disease status. Mortality rate in our study (5.5%) is much lower when compared to the other report from Iran (18.7%) [4]. We established that most of the deceased patients suffered from SCID^9^; however, our study included few cases of SCID^9^ and other severe PID cases leading to death. We also observed that most of the affected patients are from low socioeconomic status. This might be due to a lower level of education and not being aware of the risks associated with consanguineous marriage. Other studies indicated that lack of accurate clinical PID diagnostic criteria, unawareness of general practitioners and lack of referring to physicians in cases with mild presentations cause PIDs not to be discovered properly. Societies with advanced social awareness report statistics closer to the actual quantities. Therefore, long-term projects for improving social knowledge about primary immunodeficiency diseases, their clinical presentations, consanguineous marriage and its genetic effect on occurrence of PIDs are planned. We began our cooperation with the Standing Committee of Public Health in the International Federation of Medical Students Association. This standing committee is responsible for raising awareness regarding global public health issues. As a rule, the earlier the diagnosis is made, the sooner the treatment can commence and the lower morbidity and mortality can be expected.

## Conclusion

Although a national registry has been established in Iran (IPIDR) [4], this is the first comprehensive biobank for PID patients in our country, which offers cross-country collaborations with the goal of identifying genetic diagnosis of these patients. Genome sequencing and definite diagnosis may help patients to receive effective treatments and enjoy higher survival. Our study was designed to provide the PID Biobank (PIDB) in order to have a high quality DNA reservoir of these patients, shareable for international diagnostic and therapeutic collaborations. We are keen to link with other international research centers in order to share data and samples. High prevalence of consanguinity makes PIDB samples valuable for collaborative projects. This article emphasizes on raising the awareness of society and general practitioners in order to achieve timely diagnosis of these patients and prevent current mismanagements.

## Abbreviations

CVID^1^: common variable immunodeficiency
CGD^2^: chronic granulomatous disease
CMC^3^: chronic mucocutaneous candidiasis
MSMD^4^: mendelian susceptibility to mycobacterial diseases
JAGN1^5^: Jagunal homologue 1
STK4^6^: serine threonine kinase 4
CID^7^: combined immunodeficiency
USIDNET^8^: United States immunodeficiency network
SCID^9^: severe combined immunodeficiency
BTK deficiency^10^: Bruton’s Tyrosine kinase deficiency
WAS^11^: Wischott Aldrich Syndrome
IPEX^12^: Immunodysregulation polyendocrinopathy enteropathy X-linked syndrome

## References

[1] Chapel H (2012). Classification of primary immunodeficiency diseases by the International Union of Immunological Societies (IUIS) Expert Committee on Primary Immunodeficiency 2011. Clin Exp Immunol.

[2] RAPID: resource of asian primary immunodeficiency disease. http://web16.kazusa.or.jp/rapid. Accessed 15 Jan 2015. 10.1093/nar/gkn682 PMC2686530 18842635

[3] Rezaei N, Mohammadinejad P, Aghamohammadi A (2011). The demographics of primary immunodeficiency diseases across the unique ethnic groups in Iran, and approaches to diagnosis and treatment. Ann N Y Acad Sci.

[4] Aghamohammadi A, Mohammadinejad P, Abolhassani H, Mirminachi B, Movahedi M, Gharagozlou M, Parvaneh N, Rezaei N (2014). Primary immunodeficiency disorders in Iran: update and new insights from the third report of the national registry. J Clin Immunol.

[5] Al-Herz W, Aldhekri H, Barbouche MR, Rezaei N (2014). Consanguinity and primary immunodeficiencies. Hum Hered.

[6] Bousfiha AA, Jeddane L, El Hafidi N, Benajiba N, Rada N, El Bakkouri J, Kili A, Benmiloud S (2014). Moroccan society for primary immunodeficiencies (MSPID). First report on the Moroccan registry of primary immunodeficiencies: 15 years of experience (1998–2012). J Clin Immunol.

[7] Saadat M, Ansari-Lari M, Farhud DD (2004). Consanguineous marriage in Iran. Ann Hum Biol..

[8] Bittles A (2001). Consanguinity and its relevance to clinical genetics. Clin Genet..

[9] Jaber L, Halpern GJ, Shohat M (1998). The impact of consanguinity worldwide. Commun Genet..

[10] Riegman PH, Dinjens WN, Oosterhuis JW (2007). Biobanking for interdisciplinary clinical research. Pathobiology.

[11] Shickle D, Griffin M, El-Arifi K (2010). Inter- and intra-biobank networks: classification of biobanks. Pathobiology.

[12] Ransohoff DF, Gourlay ML (2010). Sources of bias in specimens for research about molecular markers for cancer. J Clin Oncol.

[13] Lippi G, Chance JJ, Church S, Dazzi P, Fontana R, Giavarina D, Grankvist K (2011). Preanalytical quality improvement: from dream to reality. Clin Chem Lab Med.

[14] Vaught J, Kelly A, Hewitt R (2009). A review of international bio banks and networks: success factors and key benchmarks. Biopreserv Biobank..

[15] Al-Herz W, Bousfiha A, Casanova JL, Chatila T, Conley ME, Tang ML (2014). Primary immunodeficiency diseases: an update on the classification from the international union of immunological societies expert committee for primary immunodeficiency. Front Immunol.

[16] Conley ME, Notarangelo LD, Etzioni A (1999). Diagnostic criteria for primary immunodeficiencies. Representing PAGID (Pan-American Group for Immunodeficiency) and ESID (European Society for Immunodeficiencies). Clin Immunol..

[17] Nederhand RJ, Droog S, Kluft C, Simoons ML, de Maat MP (2003). Investigators of the EUROPA trial. Logistics and quality control for DNA sampling in large multicenter studies. J Thromb Haemost.

[18] Vaught J, Lockhart NC (2012). The evolution of biobanking best practices. Clin Chim Acta.

[19] The International Complication of Human Subjects Research protections. 2012. http://www.hhs.gov/ohrp/international/intcomplication/intcomplication.html. Accessed 20 Jan 2015.

[20] Hansson MG (2011). The need to downregulate: a minimal ethical framework for biobank research. Methods Mol Biol.

[21] Chalmers D (2011). Genetic research and biobanks. Methods Mol Biol.

[22] Tasse AM, Budin-Ljosne I, Knoppers BM, Harris JR (2010). Retrospective access to data: the ENGAGE consent experience. Eur J Hum Genet.

[23] Gurwitz D, Fortier I, Lunshof JE, Knoppers BM (2009). Research ethics. Children and population biobanks. Science.

[24] Brothers KB, Clayton EW (2009). Biobanks: too long to wait for consent. Science.

[25] Kaufman DJ, Murphy-Bollinger J, Scott J, Hudson KL (2009). Public opinion about the importance of privacy in biobank research. Am J Hum Genet.

[26] Greely HT (2007). The uneasy ethical and legal underpinnings of large-scale genomic biobanks. Annu Rev Genomics Hum Genet.

[27] Hansson MG (2009). Ethics and biobanks. Br J Cancer.

[28] NCI Best Practices for Biospecimen Resources. Table of contents 2011. http://biospecimens.cancer.gov/bestpractices/toc/, ISBER best practices for repositories. Cell Preserve Tech. 2008; 6:1–58. Accessed 20 Jan 2015.

[29] Wang LL, Jin YY, Hao YQ, Wang JJ, Yao CM, Wang X, Cao RM, Zhang H, Chen Y, Chen TX (2011). Distribution and clinical features of primary immunodeficiency diseases in Chinese children (2004–2009). J Clin Immunol.

[30] Savage CJ, Vickers AJ (2009). Empirical study of data sharing by authors publishing in PLoS journals. PLoS ONE.

[31] Boztug K, Järvinen PM, Salzer E, Racek T, Mönch S, Garncarz W, Gertz EM, Klein C (2014). JAGN1 deficiency causes aberrant myeloid cell homeostasis and congenital neutropenia. Nat Genet.

[32] Abdollahpour H, Appaswamy G, Kotlarz D, Diestelhorst J, Beier R, Schäffer AA (2012). The phenotype of human STK4 deficiency. Blood.

[33] Leiva LE, Zelazco M, Oleastro M, Carneiro-Sampaio M, Condino-Neto A, Costa-Carvalho BT (2007). Latin American group for primary immunodeficiency diseases. Primary immunodeficiency diseases in Latin America: the second report of the LAGID registry. J Clin Immunol.

[34] Grimbacher B; ESID Registry Working Party (2014). The European society for immunodeficiencies (ESID) registry 2014. Clin Exp Immunol.

[35] Edgar JD, Buckland M, Guzman D, Conlon NP, Knerr V, Bangs C (2014). The United Kingdom Primary Immune Deficiency (UKPID) Registry: report of the first 4 years’ activity 2008–2012. Clin Exp Immunol.

[36] Ludviksson BR, Sigurdardottir ST, Johannsson JH, Haraldsson A, Hardarson TO (2014). Epidemiology of primary immunodeficiency in Iceland. J Clin Immunol.

[37] Noh LM, Nasuruddin BA, Abdul Latiff AH, Noah RM, Kamarul Azahar MR, Shahnaz M, B H O A (2013). Clinical-epidemiological pattern of primary immunodeficiencies in Malaysia 1987–2006: a 20 year experience in Four Malaysian Hospitals. Med J Malaysia.

[38] Rhim JW, Kim KH, Kim DS, Kim BS, Kim JS, Kim CH, Kim HM, Park HJ, Pai KS, Son BK (2012). Prevalence of primary immunodeficiency in Korea. J Korean Med Sci.

[39] Kindle G, Gathmann B, Grimbacher B (2014). The use of databases in primary immunodeficiencies. Curr Opin Allergy Clin Immunol.

[40] Ehlayel MS, Bener A, Laban MA (2013). Primary immunodeficiency diseases in children: 15 year experience in a tertiary care medical center in Qatar. J Clin Immunol.

[41] Al-Tamemi S, Elnour I, Dennison D (2012). Primary immunodeficiency diseases in Oman: 5 years’ experience at sultan qaboos university hospital. World Allergy Organ J..

[42] Kilic SS, Ozel M, Hafizoglu D, Karaca NE, Aksu G, Kutukculer N (2013). The prevalences [correction] and patient characteristics of primary immunodeficiency diseases in Turkey–two centers study. J Clin Immunol.

[43] Al-Herz W (2008). Primary immunodeficiency disorders in Kuwait: first report from Kuwait National Primary Immunodeficiency Registry (2004–2006). J Clin Immunol.

